# Supplementary material for: Spiroplasma endosymbiont reduction of host lipid synthesis and Stomoxyn-like peptide contribute to trypanosome resistance in the tsetse fly Glossina fuscipes
Source: bioRxiv. 2024 Oct 24:2024.10.24.620045. Preprint. [Version 1] doi: 10.1101/2024.10.24.620045 (PMC11527105; doi:10.1101/2024.10.24.620045)
Supplement: Supplement 1 [file NIHPP2024.10.24.620045v1-supplement-1.pdf]

bioRxiv preprint doi: https://doi.org/10.1101/2024.10.24.620045; this version posted October 24, 2024. The copyright holder for this preprint (which was not certified by peer review) is the author/funder, who has granted bioRxiv a license to display the preprint in perpetuity. It is made available under aCC-BY 4.0 International license.

Supplementary data

Fig. S1. Overview of the *Gff* Transcriptome Study.

A. Table summarizes results obtained from different biological replicates across three conditions, *Ctrl*, *Spi*$^+$ and *Tpi*$^+$.

Condition: Ctrl: *Spiroplasma* and trypanosome negative midgut; *Spi*$^+$: *Spiroplasma* positive midgut; *Tpi*$^+$: trypanosome positive midgut; BRep: Biological Replicate; UMR: Number of Uniquely Mapped Reads to the *Gff* genome (*Gff*_genome-2018_ver 63); No. Trans: Number of expressed transcripts, defined as those with normalized read coverage ≥ 10 in at least 50% of the biological replicates per condition.

B. Heat map showing the Euclidean distances between biological replicates, calculated from the regularized log transformation of the data, provides insights into the similarities and differences among the conditions.

Fig. S2. Differentially Expressed Shared Transcripts and Peritrophic Matrix Integrity.

The Heat map denotes the fold changes of differentially expressed (DE) genes that are shared between the *Spi*$^+$ and *Tpi*$^+$ states, according to their putative functions. Fold-change values are expressed as a fraction of the average normalized gene expression levels from age-matched *Spi*$^+$ or *Tpi*$^+$ relative to the control *Ctrl*. The heat maps (dendrograms) were generated using Euclidean distance calculation combined with ward.D clustering methods within the R-package software. The clusters were manually separated into two categories: PM and Immunity Functions.

bioRxiv preprint doi: https://doi.org/10.1101/2024.10.24.620045; this version posted October 24, 2024. The copyright holder for this preprint (which was not certified by peer review) is the author/funder, who has granted bioRxiv a license to display the preprint in perpetuity. It is made available under aCC-BY 4.0 International license.

B. Effect of *Spiroplasma* infection on Peritrophic Matrix integrity. The survival of flies was monitored every 48 hours following a *per os* treatment of teneral adult flies with *Serratia marcescens*, administered 72 hours post-eclosion. At time of death, the *Spiroplasma* infection status of each fly was evaluated using our diagnostic assay. The Kaplan-Meyer survival curves illustrate the fly survival over time for *Spiroplasma*-uninfected flies (blue) and *Spiroplasma*-infected flies (red). This experiment was conducted twice, with no significant differences observed between the two experiment (Data not shown).

Fig. S3. Heatmap representation of unique differentially expressed (DE) transcripts.
The heatmaps depict the fold changes of unique DE transcripts across various functional categories, comparing infected transcriptomes and the uninfected control. *Spi*+: *Spiroplasma* infected, *Tpi*+: trypanosome infected; FC: fold change indicate the degress of change in expression levels relative to uninfected controls; 1: differentially expressed transcripts; NDE: transcripts that are not differentially expressed.

Fig. S4. Genomic Characterization of Stomoxyn locus.
A. Phylogenetic tree of mature Stomoxyn sequences from nine different Diptera species based the Maximum likelihood (ML) model. Sequences used in this analysis were obtained from VectorBase for *S. calcitrans* (*Scal*Stomoxyn; SCAU016937 and *Scal*Stomoxyn 2; SCAU016907), *Gff (Gff*Stomoxyn-like*;* GFUI18_001176*)*, *Gpp (Gpp*Stomoxyn*; GPPI027903*) and *M. domestica* (*Mdom*Stomoxyn-like*; MDOA008330*)*,* and from NCBI database for *L. cuprina* (*Lcup*Stomoxyn*;* KAI8119624.1 and *Lcup*Stomoxyn-like*;* XP_023308701.2)*, S. bullata* (*Sbul*Stomoxyn; DOY81_004902), *L. sericata* (*Lser*stomoxyn-like*;* XP_037825072.1), *Episyrphus balteatus* (*Ebal*Stomoxyn-like; XP_055851874.1) and *Eupeodes corollae* (*Ecor*Stomoxyn-like; XP_055904620.1). The analysis involved 11 amino acid sequences and 1000 bootstrap replications. **B.** Genomic content surrounding the Stomoxyn-like gene (GFUI18_001176) focusing on the supercontig JACGUE010000004 of the Gff genome assembly (version 63, Vectorbase). The supercontigs available from the other *Glossina* WGS data were also compared. Genes exhibiting synteny among different tsetse species are indicated by arrows with the same color genes that do not shown synteny are presented in gray. The black arrow marks the expected location of the *Stomoxyn* gene in *G. pallidipes, G. austeni* and *G. brevipalpis*. Gene size and spacings are not drawn to scale. Based on the current assembly of the region, only one *Stomoxyn*

bioRxiv preprint doi: https://doi.org/10.1101/2024.10.24.620045; this version posted October 24, 2024. The copyright holder for this preprint (which was not certified by peer review) is the author/funder, who has granted bioRxiv a license to display the preprint in perpetuity. It is made available under aCC-BY 4.0 International license.

gene is present in *Gff* and *Gpp,* while it is absent in other *Glossina* species. **C.** Multiple sequence alignment of the Stomoxyn locus from *Gff*, *Gpp* and *Gpg.* The alignment includes genomic PCR product sequences of the *Stomoxyn* locus from flies obtained from laboratory and field populations, confirming the presence and conservation of this locus in the species from Palpalis subgroup. The primers for the PCR amplification were designed to span the entire coding region of the Stomoxyn pre-pro-mature peptide.

Fig. S5. Expression of *attacin* and *cecropin* in *Tpi*$^{+}$ flies 15 days post emergence.
Flies were exposed to a bloodmeal spiked with BSF *Tbb* in their first meal and fifteen days later, their midguts were dissected 48 hours after their last bloodmeal. A control group of flies were treated similarly but received normal blood meals without any parasites. *Trypanosome* and *Spiroplasma* infection status of each fly was determined as described in materials and methods section. A total of eleven biological replicates were analyzed for *Ctrl* and eight for *Tpi*$^{+}$ groups. The expression of *attacin* and *cecropin* were quantified using RT-qPCR relative to tsetse *gapdh*. Expression levels showed significant difference (p=0.001) between the *Tpi*$^{+}$ and *Ctrl* groups.

Fig. S6. Bioactivity of Stomoxyn Against Trypanosomes. A. *In vitro* killing activity of rec*Gff*Stomoxyn against mammalian BSF trypanosomes **B.** *In vitro* killing activity of rec*Gff*Stomoxyn against insect specific procyclic PCF trypanosomes. Data shown represent findings from the second replicate experiment showing the potency of *Gff*Stomoxyn against BSF and PCF forms.

Table S1. PCR primers used in this study.

Table S2. Detailed results and analysis of each transcriptome.
